# COVID-19, climate change, and communities

**DOI:** 10.1016/S2542-5196(21)00257-6

**Published:** 2021-10-07

**Authors:** Emma Sacks, Sonam Yangchen, Robert Marten

**Affiliations:** a Department of International Health, Johns Hopkins School of Public Health, Baltimore, MD 21205, USA; a Alliance for Health Policy and Systems Research, World Health Organization, Geneva, Switzerland

The ongoing COVID-19 pandemic exposes most countries’ weak public health systems and poor preparedness for disasters. The pandemic also highlights the crucial importance of community leadership. Alongside rapid action from governments, responding to a crisis requires massive local efforts to provide food, supplies, and medical care, as well as organise mutual aid, education campaigns, activism, and support networks. The pandemic shows how and why communities must be engaged and empowered not only to respond, but also to prevent and plan for adaptation to climate change. Given the profoundness of the looming climate crisis—one that will lead to more regional and global environmental and health catastrophes—learning lessons from the COVID-19 pandemic and developing better approaches for promoting community leadership is crucial.

As climate change continues to destroy habitats and livelihoods, infectious diseases will spread more easily and rapidly through populations.^1^ Climate and climate-related events have contributed to doubling the number of refugees in the past decade, and in the coming years more people will be forced to leave their homes and face extreme weather.^2^ Governments have been slow to act, and few have treated climate change as a crisis requiring large-scale response. Indeed, in February, 2021, an interim assessment of countries’ pledges to cut greenhouse gases shows that most countries are making woefully insufficient progress.^3^ Populations most likely to experience negative climate change effects are those in low-income and middle-income countries, including those in small island states or coastal communities, Indigenous groups, and those in precarious economic situations. Yet, these constituencies are often not considered or are ignored by policy makers.^4^

Climate change, and its impacts on equitable access to health services, has been largely overlooked within the public health community. Despite substantial effects on health systems, health policy makers and researchers rarely consider how to reduce, mitigate, or respond to climate change.^5^ A recently released global roadmap for health care decarbonisation shows various ways to advance efforts for reducing worldwide emissions in health care.^6^ Health systems must find ways to promote clean energy use, reduce carbon in supply chains, and facilitate sustainable travel, but there is also a crucial need to do more to engage multisectorally with policy processes beyond the health sector.

Community-based strategies have been shown to be effective in addressing priority health issues, especially when integrated into broader local and national health strategies.^7^ One of two carbon-neutral countries, Bhutan implemented a strategy for engaging communities for prevention and control of pandemics, identifying champions in each community and establishing community support systems. Village health workers were trained across the country to provide surveillance on COVID-19, and volunteers and community organisations engaged in logistics management, surveillance, and delivery of essential items, ensuring that vulnerable populations were not excluded.^8^

In disaster preparedness and response, governments must empower and enable health systems to work with communities to identify priorities, strategies, and solutions. One way to implement existing approaches, as well as identify new solutions for health system reform, is by co-producing knowledge, with particular attention to working with communities.^9^ This approach demands not only action at the national level, but also subnational and local engagement, to identify leaders among affected and vulnerable communities. As was also shown during the Ebola crisis, the COVID-19 pandemic highlights the limitations of top-down approaches. Although there is no universal agreement on the role of communities in health systems,^10^ the literature is rich in examples of the positive health effects of community participation and mobilisation.^7^ Community members and organisations, including Indigenous groups, are particularly well suited to act as the frontline response in dynamic crises and health emergencies but must be supported and encouraged to participate in these processes (panel).

Panel **Examples of the roles of communities for pandemic prevention, preparedness, mitigation, and response via their ability to harness local knowledge and awareness**

**Specific skill sets of community members and organisations**

• Might be attuned to the particular dynamic epidemiological conditions
• Can speak the local language
• Have familiarity with customs and practices of the region
• Extant organisations and organisations might be already advocating for climate mitigation strategies and planning for disaster response
• Trusted community members might be able to link facilities, politicians, and community members or organisations

**Strategies for increased community involvement**

• Ensure that stakeholder meetings are held in multiple languages and in accessible locations
• Identify local leaders and existing community organisations already involved in disaster preparation and response or adjacent areas
• Include community members in message planning, especially in developing health and safety messages with empathy and no judgment
• Work with community members to lead strategy and co-produce priority-setting agendas
• Include community organisations in policy, budget, and implementation processes from the beginning

For preparedness and response strategies to be effective, there must be better efforts to facilitate community engagement and leadership. Power dynamics must be recognised and understood to ensure that existing divisions in communities are not exacerbated and that those who are most marginalised are given opportunities to participate. Communities must be involved in climate change prevention and mitigation strategies, disaster preparedness planning, as well as immediate response and implementation strategies. The only way to reduce climate disaster and respond in an equitable way is for communities to take the lead. Communities can plan, adapt, and respond to emergencies, but must be given the opportunity and resources to lead.

We declare no competing interests.
